# Advances in Soil Amendments for Remediation of Heavy Metal-Contaminated Soils: Mechanisms, Impact, and Future Prospects

**DOI:** 10.3390/toxics12120872

**Published:** 2024-11-29

**Authors:** Xinyi Nie, Xianhuai Huang, Man Li, Zhaochi Lu, Xinhe Ling

**Affiliations:** 1 School of Environment and Energy Engineering, Anhui Jianzhu University, Hefei 230601, China; 2 Anhui Provincial Key Laboratory of Environmental Pollution Control and Resource Reuse, Hefei 230601, China; 3 Institute of Geotechnical Engineering, Southeast University, Nanjing 211189, China; 4 College of Civil Engineering, Anhui Jianzhu University, Hefei 230601, China

**Keywords:** heavy metal, soil degradation, contaminated soils, soil amendments, bibliometric analysis

## Abstract

Heavy metal contamination is a critical factor contributing to soil degradation and poses significant environmental threats with profound implications for ecosystems and human health. Soil amendments have become an effective strategy to address these challenges by reducing heavy metal hazards and remediating contaminated soils. This review offers a comprehensive analysis of recent advancements in soil amendments for heavy metal-contaminated soils, with a focus on natural, synthetic, natural-synthetic copolymer, and biological amendments. By thoroughly examining and contrasting their remediation mechanisms and effects, this study provides a detailed evaluation of their influence on soil physicochemical properties, leachable heavy metal content, and microbial communities. Through bibliometric analysis, current research priorities and trends are highlighted, offering a multidimensional comparison of these amendments and clarifying their varying applicability and limitations. Furthermore, this review explores future prospects and the inherent challenges in soil amendments for heavy metal contamination, aiming to offer valuable insights and theoretical references for the development and selection of novel, efficient, multifunctional, environmentally friendly amendments.

## 1. Introduction

Heavy metals are released into the soil through both natural and anthropogenic sources [1]. With increasing levels of industrialization and the continuous development of transportation networks worldwide, industries such as metallurgy, mining, construction materials manufacturing, and transportation generate a large amount of industrial waste containing heavy metals. This has led to the entry of heavy metals such as lead (Pb), mercury (Hg), chromium (Cr), and cadmium (Cd) into the atmosphere, water, and soil, causing serious environmental pollution [2]. Industrial waste, agricultural fertilizers, spills from petroleum products, coal combustion, livestock manure, wastewater irrigation, and urban sewage sludge are all important sources of heavy metals in contaminated soils [3].

Unlike other organic compounds, heavy metals are not easily purified through natural processes, leading to their accumulation in the environment. This accumulation can have adverse effects on the biological community, including changes in microbial communities, accumulation in plants and soil organisms, and risks to animal, plant, and human health [4]. The results of cancer risk studies show that approximately 76% and 15.7% of cancer risks are caused by high levels of Cd and arsenic (As), respectively [5]. Consuming rice grown in As, Cd, and Cr-contaminated fields significantly raises the risk of cancer. Mercury deposited in land and water can form more toxic methylmercury, damaging the central nervous system and causing blindness, deafness, and other symptoms [6]. The gradual accumulation of heavy metals in the soil leads to increasingly severe soil degradation phenomena, such as weak soil structure, susceptibility to erosion, nutrient loss in plants, and decreased soil biodiversity [7].

The excessive accumulation of heavy metals in the soil can alter the physical and chemical properties of the soil. Pearson correlation analysis demonstrated that soil pH, CaCO_3_, and clay content are critical factors influencing the binding forms and types of heavy metals in soil fractions, thereby significantly affecting their bioavailability and chemical reactivity [8,9]. Heavy metal toxicity disrupts microbial communities by inhibiting their growth and metabolic activities, reducing diversity and ecological function. This disruption and altered enzyme activities due to heavy metal interference impair organic matter decomposition and nutrient cycling. Consequently, these changes adversely affect soil structure, leading to reduced porosity, compressive strength, and water-holding capacity, further degrading soil health [3].

Soil contaminated with heavy metals has a detrimental impact on human health and the ecological environment [1]. Remediating heavy metal-contaminated soil is a growing area of research. Techniques involve physical, chemical, and biological methods to reduce pollutant concentrations, immobilize them, and convert them into less harmful forms, preventing their spread in the ecosystem [1,3,10,11,12,13]. Approaches for remediating heavy metal-contaminated sites are generally categorized into in situ and ex situ technologies. In situ methods include electrokinetic separation, soil amendment, thermal treatment, in situ chemical leaching, chemical oxidation-reduction, solidification/stabilization, phytoremediation, nanotechnology, and microbial remediation [1,3,14,15,16,17]. Ex situ technologies comprise attrition scrubbing, landfilling, chemical leaching, thermal desorption, co-disposal in cement kilns, and the use of prefabricated beds [1,11,18]. The United States Environmental Protection Agency (USEPA) analyzed the remediation technologies utilized at 106 sites, which were documented in 172 source decision documents from the Superfund program from 2018 to 2020 [19]. Table 1 summarizes the specific types of soil remediation techniques selected in these documents. Of the 48 (37%) documents designated for treatment, in situ remediation technologies accounted for 28% of the total projects, while ex situ remediation technologies made up 13%. The most selected in situ technologies for soil remediation in recent years include in situ thermal treatment (ISTT), soil vapor extraction (SVE), solidification/stabilization (S/S), and chemical treatment methods, which encompass in situ chemical oxidation (ISCO) and in situ chemical reduction (ISCR). For ex situ soil treatment, S/S and physical separation are the most frequently selected remedies.

When selecting soil remediation techniques for different types of soil pollution, factors such as remediation objectives, socio-economic conditions, pollution types, pollution levels, remediation time, and feasibility of the techniques must be considered [11]. Additionally, soil remediation techniques for heavy metal-contaminated sites can be categorized into physical, chemical, and biological techniques based on their operational principles [1,3,11,12,13]. Some of these techniques require the use of soil amendments, such as weak acids and acid salts, to remove heavy metals [14,20,21,22].

**Figure 1:**
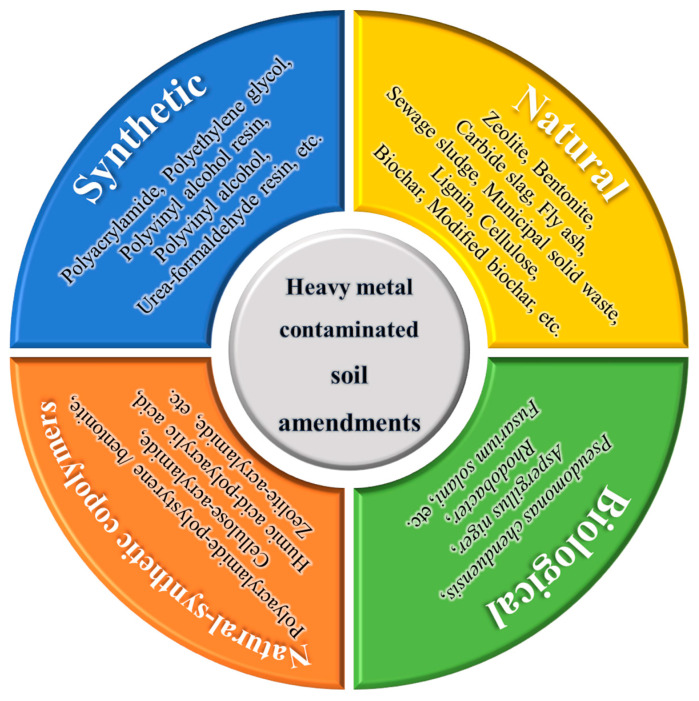
Classification system chart of heavy metal contaminated soil amendments [10].

Soil amendments can be categorized into four main groups based on the source of raw materials—natural, synthetic, natural-synthetic copolymers, and biological amendments. Representative examples in each category are summarized as shown in Figure 1 [10]. Table 2 presents a selection of typical soil amendments used for heavy metal soil remediation. The soil amendments are organized by classification, experiment types, types of heavy metals in contaminated soils, and remediation effectiveness, along with their corresponding references. However, both the nature of the amendment and its application have limitations in remediating heavy metal-contaminated soils. For instance, industrial solid wastes such as red mud, fly ash, and calcium carbide slag may contribute to secondary pollution in these soils. Organic amendments can either release nitrogen, reducing the soil’s capacity to retain organic carbon, or contain high levels of pathogens, parasites, and toxic substances, which can adversely affect plants and soil organisms to varying degrees. Excessive application of organic amendments, particularly biochar, can reduce soil nitrogen availability by increasing microbial nitrogen demand, raising the carbon–nitrogen ratio, and temporarily fixing nitrogen, ultimately leading to nitrogen deficiency [23]. However, more research is needed on the types and amounts of these combinations [24]. 

In this review, we aim to: (1) comprehensively analyze recent advances in soil amendments for heavy metal-contaminated soils, focusing on natural, synthetic, natural-synthetic copolymers, and biological amendments; (2) investigate and compare their remediation mechanisms thoroughly, as well as their impacts on soil physicochemical properties, leachable heavy metal contents, and microbial communities; (3) highlight current research focuses and trends through bibliometric analyses relevant to the soil heavy metal remediation, providing multidimensional comparisons of these amendments and elucidating their varying applications and limitations; and (4) explore future prospects and challenges for the development and selection of novel, highly efficient, and multifunctional environmentally friendly soil amendments.

## 2. Bibliometric Analysis of Hotspots and Frontiers on Remediation Materials for Heavy Metal-Contaminated Soils

Using the Thomson Reuters Web of Science (WOS) database, the search period was 2013–2023, and the search formula was Topic = (heavy metal soil) and (soil remediation) and (soil amendments) and (materials), and a total of 317 articles were retained in the Web of Science Core Collection as a data sample for systematic analysis through screening and de-duplication to remove irrelevant and duplicated literature. Using CiteSpace software (version 6.16) and Origin 2022 software, the relevant literature published in the last 11 years was analyzed econometrically. 

**Figure 2:**
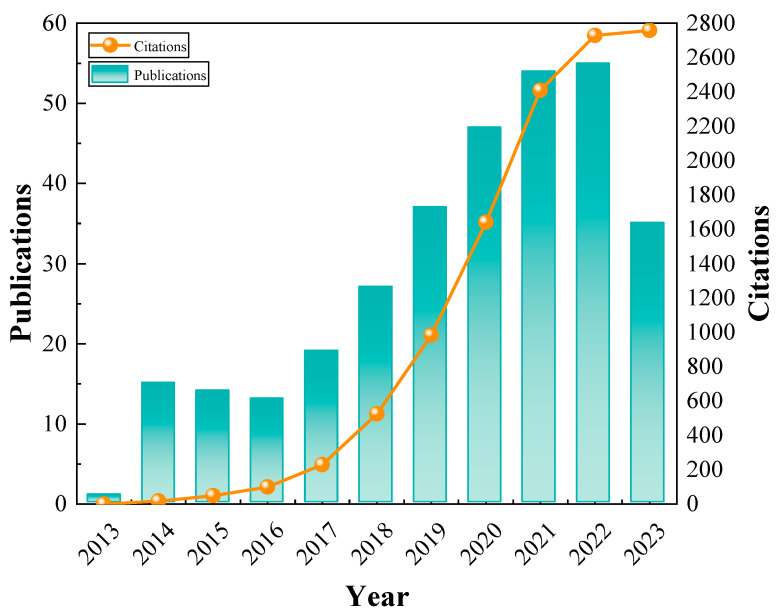
The distribution of citations and publication number by year.

Figure 2 shows publication number and citation count trends. The number of publications is a key indicator of research interest in a field. The citations in Figure 2 represent the total number of citations to articles on heavy metal-contaminated soil amendments from 2013 to that year. The greater the slope of the line, the more citations there were that year. The bar chart in Figure 2 illustrates the number of publications each year. From 2013 to 2016, the number of publications and citations in the relevant fields was low. During the period 2017–2022, the publication and citation number increased rapidly, indicating the increasing demand for remediation of heavy metal-contaminated soils.

### 2.1. Country Co-Authorship Analysis

**Figure 3:**
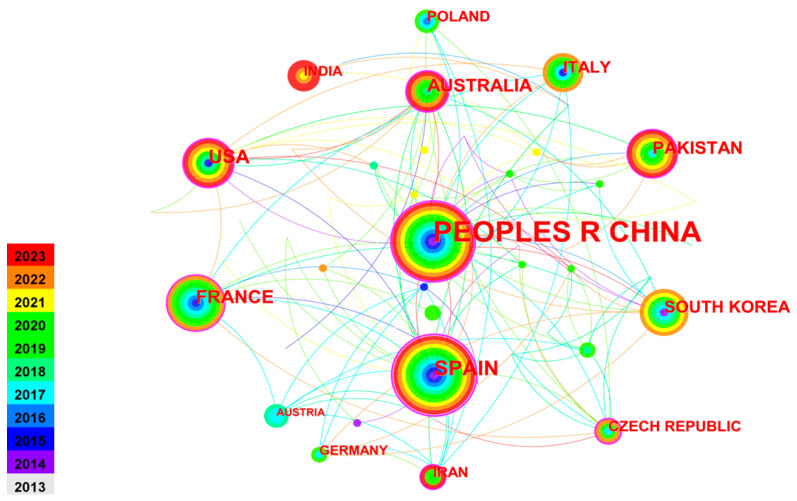
Cooperation network of productive countries from 2013 to 2023.

Figure 3 depicts the collaborative network among 61 countries researching soil remediation for heavy metals, with 133 collaborative links. Table 3 ranks the top 10 countries by publication count. China leads with 155 papers, followed by Spain (35), USA (25), France (21), and Australia (20), accounting for 78.29% of the total. These countries have made significant contributions to the field. China’s high publication count suggests a strong focus on this area. Spain, China, the USA, and Australia have higher centrality, indicating greater influence. Extensive interconnections in the visualization suggest active communication and collaboration between researchers worldwide.

### 2.2. Keyword Co-Occurrence Analysis

**Figure 4:**
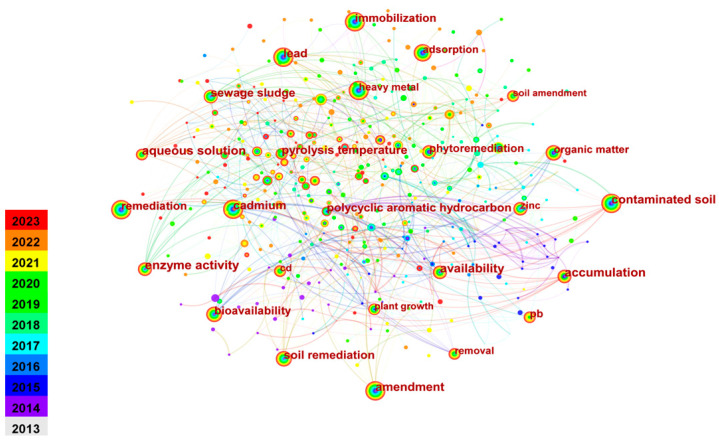
A network of keyword co-occurrence from 2013 to 2023.

The frequency of keywords in a specific period reflects research hotspots. Emergence analysis with a 1-year time slice using CiteSpace software reveals research progress and cutting-edge hotspots. Figure 4 displays keyword co-occurrence in heavy metal-contaminated soil amendments from 2013 to 2023, while Table 4 ranks the top 10 keywords based on centrality. “Stabilization” has the highest occurrence frequency after “Heavy metal”, with related terms like “Immobilization”, “Stabilization/solidification”, and “Solidification/stabilization” also in the top ten, indicating a focus on solidification remediation. 

Table 5 presents the burst intensity, start and end times, and duration of these keywords. The interval time period is marked by blue lines, while the burst time period of the keywords is indicated by red lines. The red timeline highlights bursts corresponding to highly cited keywords [34]. Table 5 highlights the emergence analysis of keywords in heavy metal-contaminated soil remediation materials from 2013 to 2023. From 2013 to 2018, initial interest in “organic amendment”, “mine tailing”, and “black carbon” waned, possibly due to limited effectiveness and concerns about secondary pollution. Between 2021 and 2023, “pollution” and “black carbon” became the most popular amendments, reflecting a growing concern about secondary pollution and health risks. 

**Figure 5:**
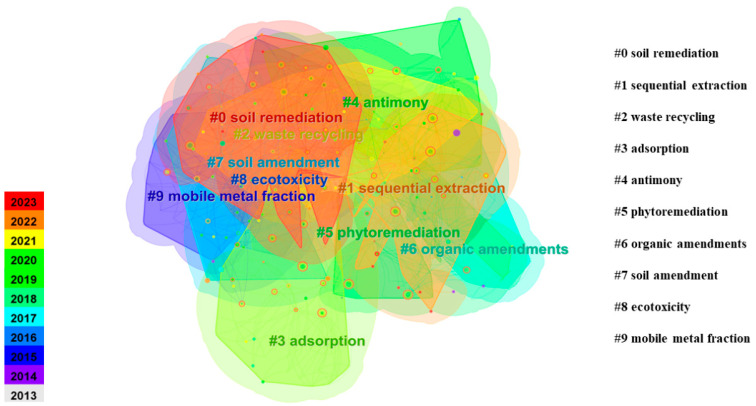
A cluster map of co-cited literature on heavy metal-contaminated soil remediation from 2013 to 2023.

Keyword clustering analysis is a useful technique for presenting important research findings. Table 6 and Figure 5 provide information about the keyword clusters and their associated features. Ten clusters of basic keywords are shown based on graphical and chronological order, where #0 is the largest clustering. The mean silhouette (S) and modularity (Q) are two crucial metrics for evaluating the quality of co-cited node clusters, reflecting the “overall structural characteristics” of the clusters within the co-cited network, which can range from 0 to 1. Higher values of Q signify more effective node clusters, with a Q value greater than 0.3 suggesting a notable community structure in the network. Similarly, increased S values indicate greater uniformity among the nodes in a cluster, and typically, an S value exceeding 0.7 implies that the cluster is highly credible. In this research, Q and S are 0.498 and 0.739, respectively, which indicates that our network structure has a significant community structure and node reliability. The year represents the median year of all the references within this cluster. Log-likelihood ratio (LLR) is a statistical method used to identify and analyze relationships between nodes in a network, and CiteSpace divided these subject categories into 10 clusters using the LLR algorithm [35].

The keyword co-occurrence network diagram for the remediation of heavy metal-contaminated soil is shown in Figure 5. The size of each node corresponds to the frequency of the associated cluster, with larger nodes representing higher frequency [34]. As shown in Figure 5 and Table 4, there is a noticeable variation in the size of the clusters. The largest cluster, #0, contains 47 nodes, accounting for approximately 14.4% of the total nodes of the co-occurrence network. In contrast, cluster #9 is the smallest, with only 18 nodes representing just 5.5% of the co-citation network. From Figure 5 and Table 4, it can be observed that the five largest clusters in the heavy metal-contaminated soil remediation research network are as follows: cluster #0 “soil remediation” (47 occurrences), cluster #1 “sequential extraction” (45 occurrences), cluster #2 “waste recycling” (40 occurrences), cluster #3 “adsorption” (34 occurrences), and cluster #4 “antimony” (40 occurrences). The remaining clusters are detailed in Table 4. These terms represent the current research hotspots in the field, reflecting the prominent themes from 2013 to 2023.

**Figure 6:**
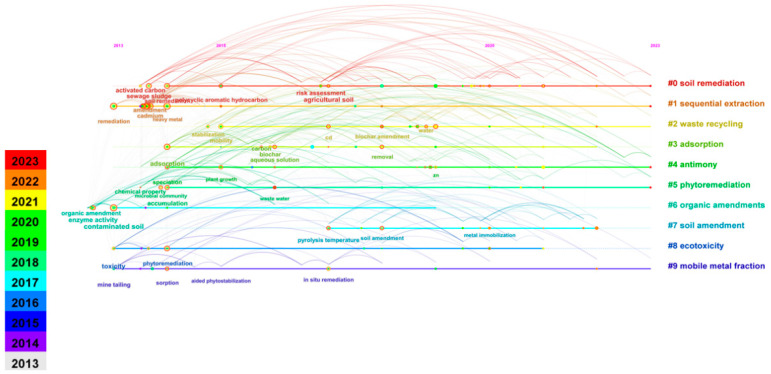
Timeline visualization of the 10 clusters of document co-citations from 2013 to 2023 based on 1-year slices.

Figure 6 presents the keyword timeline view, offering insights into the temporal distribution of keywords. The size of each node is proportional to the frequency of keyword co-occurrence, while nodes in different colors represent different years. The color of each cluster label corresponds to the average year of that cluster [35]. From Figure 6, the following conclusions can be drawn. Studies conducted between 2013 and 2016 primarily focused on organic amendments, chemical leaching, and chemical oxidation-reduction processes (clusters #3, #6, #8, #9). However, following the promulgation of policies related to soil remediation and the recognition of limitations in some chemical methods, research from 2016 to 2023 gradually shifted towards solid waste reuse and stabilization/solidification (clusters #0, #1, #2, #4, #7). Phytoremediation (cluster #5) has garnered significant attention due to its low cost and minimal environmental impact.

## 3. Natural Soil Amendments 

### 3.1. Inorganic Soil Amendments

#### 3.1.1. Natural Mineral 

Common natural minerals used for remediating heavy metal-contaminated soils primarily include clay minerals and natural zeolites. Due to their ion exchange properties, natural zeolites are highly effective at adsorbing metal cations [36]. Among natural zeolites, clinoptilolite is most commonly used for heavy metal adsorption, followed by mordenite and chabazite. Clinoptilolite, the most widely utilized, is particularly effective in removing metals such as Pb, copper (Cu), zinc (Zn), Cd, and nickel (Ni) [37]. Zeolites are porous hydrated aluminosilicates characterized by their molecular sieve properties, which arise from their well-defined network structure [38].

Clay minerals, broadly categorized as hydrous aluminosilicates, form the colloidal portion of soils, sediments, rocks, and water. Known for their strong ion exchange and adsorption capacities, these minerals are key natural adsorbents for heavy metals, playing a crucial role in the remediation of soils contaminated with such pollutants. In agricultural soil remediation, sepiolite has been the most frequently used amendment, followed by palygorskite and bentonite [39]. Bentonite minerals consist of montmorillonite units arranged in a 2:1 layered structure of silica sheets. They have a large surface area, high cation exchange capacity, and a negative charge, making them effective adsorbents [40]. Palygorskite, also known as attapulgite, is a magnesium aluminum phyllosilicate. Its surface hydroxyl groups can form inner-sphere complexes with Cd^2+^ ions, while deprotonated hydroxyl groups can create outer-sphere complexes through electrostatic binding, effectively reducing Cd availability in the soil [41].

**Figure 7:**
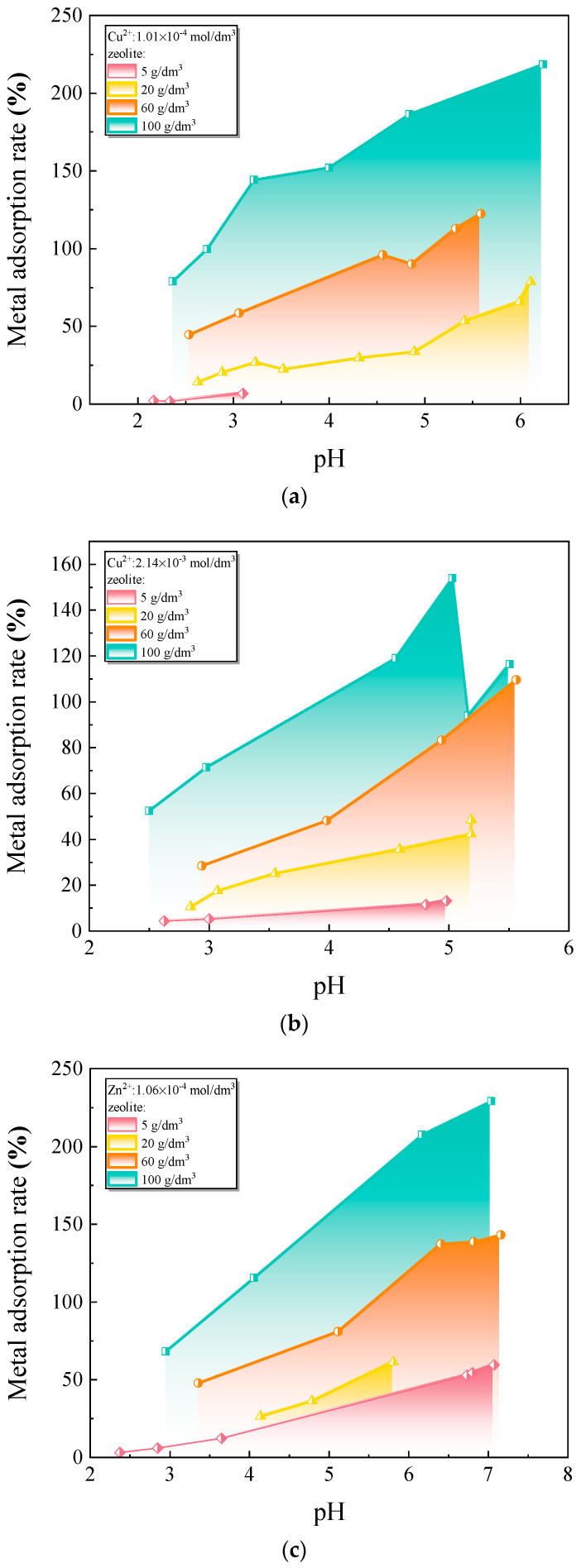

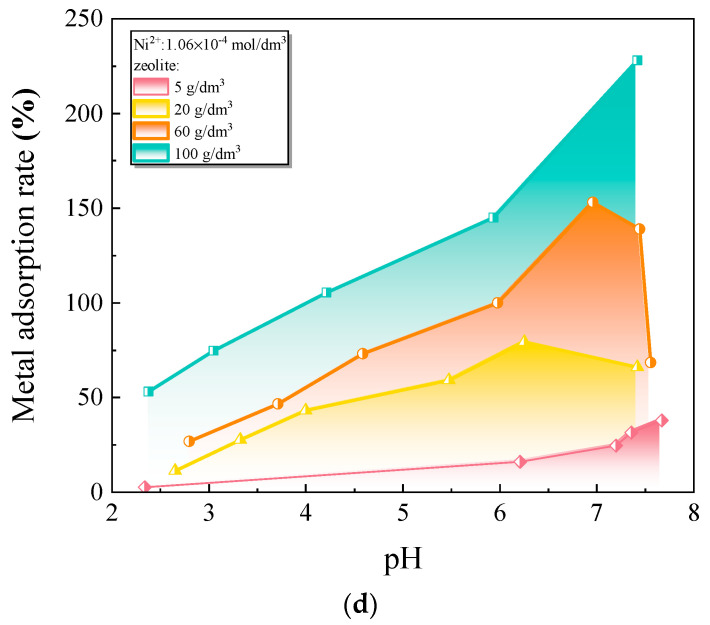
Experimental metal pH-adsorption edges at various zeolite concentrations: (**a**,**b**) Cu^2+^; (**c**) Zn^2+^; (**d**) Ni^2+^ [45].

Based on the natural mineral substance structure and adsorption principle, the remediation of heavy metal-contaminated soil mainly includes the following methods: (1) improving soil structure. Bentonite, mainly composed of montmorillonite, has certain expansion, dispersion, and adhesiveness. Adding it to the soil can increase the number of aggregates, increase soil porosity, and reduce soil bulk density [42]. (2) adjusting soil pH value. Bentonite is a porous, fibrous, hydrated magnesium silicate. Its structure consists of two tetrahedral silica sheets sandwiching an octahedral magnesium/hydroxide sheet [39]. Bentonite alters soil pH and heavy metal retention through adsorption and ion exchange mechanisms. At low pH, metal retention is mainly due to surface deprotonation and ion exchange, while at higher pH, metal ion precipitation further enhances retention, especially for Cu and Zn [41,43]. Lime, as an alkaline calcium compound, can raise the pH value of acidic soil contaminated with heavy metals through neutralization reactions and reduce the solubility and mobility of heavy metals in the soil [44]. Zeolite has the capacity to adsorb Na^+^ and Cl^−^ from the soil, which effectively reduces soil salinity and alleviates the impacts of salinization. Additionally, zeolite can buffer the pH levels of saline-alkali soils. At high pH, the primary effect is the conversion of toxic inorganic compounds into poorly soluble hydrated oxides and carbonates, thereby mitigating their potential toxicity and improving soil quality [45]. (3) adsorbing heavy metals. Additionally, zeolite is a potent natural adsorbent for heavy metals like Cu^2+^, Zn^2+^, and Ni^2+^, with adsorption efficiency influenced by soil pH. The equilibrium adsorption tests were conducted by mixing sieved and dried zeolite particles with solutions containing various heavy metals at different pH levels. The flasks were shaken at a constant temperature for a duration of four days. The residual concentration of heavy metals in the solution is measured at the end of the reaction. The difference between the initial heavy metal concentration and the residual concentration represents the quantity of heavy metals that have been adsorbed, which indicates that adjusting pH can optimize the adsorption process for practical applications. Tests indicate that zeolite’s removal of these metals depends on pH, with higher pH (around 2–3) enhancing adsorption capacity, as seen in Figure 7 [45].

Bentonite and kaolin are widely used for heavy metal adsorption, with varying effectiveness for Pb^2+^, Cu^2+^, and Zn^2+^. Higher H^+^ on clay surfaces and reduced metal ion precipitation at high pH levels decrease heavy metal retention in clay-metal systems. Clay suspension samples were prepared by conducting batch equilibrium tests, in which kaolin or bentonite was mixed with heavy metal solutions at a specified metal ion concentration. After shaking for a predetermined duration, the suspension samples were centrifuged and filtered to separate the liquid phase from the solid phase. Bentonite’s adsorption of heavy metals follows Temkin and Langmuir models over a wide concentration range, while kaolin follows Freundlich and Langmuir models. The Temkin isotherm is highly effective for predicting gas-phase equilibrium but may be less suitable for more complex adsorption systems. The isotherm can be described as Equation (1).

(1) $$Temkin:\;{S=\mathrm{Bln}(A}_{T}{)+\mathrm{BlnC}}_{e}$$

where A_T_ denotes the Temkin isotherm equilibrium binding constant (L/mM) and B (J/mol) represents the constant related to the heat of sorption. 

The Freundlich isotherm is commonly applied to multilayer adsorption on heterogeneous surfaces and can be expressed in its linearized form by Equation (2).

(2) $$Freundlich:\;{\mathrm{lnS}=\mathrm{lnK}+\mathrm{nlnC}}_{e}$$

In the equation, S (mmol/g) represents the specific adsorption coefficient of heavy metal ions per unit mass of soil. K (mmol^1−n^ g^−1^ Ln) represents the Freundlich adsorption capacity coefficient. n (dimensionless) is the adsorption constant, which measures the adsorption strength or surface heterogeneity when 0 < n < 1. When n < 1, it represents a chemical adsorption process, and when n > 1, it represents a cooperative adsorption process. C_e_ (mmol/L) represents the final concentration of heavy metal ions after 48 h of solid/liquid equilibrium.

Contrary to the Freundlich model, the Langmuir adsorption isotherm is typically used to describe monolayer adsorption and homogeneous adsorption. Its mathematical expression is shown in Equation (3) [43].

(3) $$Langmuir:\;C_{e}{/S=1/(\mathrm{bS}}_{\max}{)+(1/S}_{\max}{)C}_{e}$$

In the equation, S_max_ (mM g^−1^) represents the maximum Langmuir adsorption capacity, and b (1/Mm) represents the Langmuir model constant. 

Vermiculite is a widely available natural clay. Zhao et al. [46] conducted column immersion tests to study the effects of coexisting cations and evaluate the practical performance of vermiculite in heavy metal removal. They found that vermiculite had a good effect on removing heavy metals. Malandrino et al. [25] conducted pot experiments and demonstrated that adding vermiculite significantly reduced the absorption of metal pollutants by lettuce and spinach plants, confirming its potential for the remediation of metal-contaminated soils. Furthermore, zeolite can retain water. Adding zeolite to soil can improve its water retention capacity. It can also enhance the physicochemical properties and hydraulic parameters of the soil, thereby reducing nutrient loss [47]. 

Natural minerals encounter theoretical and technical challenges in practical use, including managing application rates, methods, and timing and limitations in available reserves for large-scale applications. Studying the ideal application rates for single amendments and the optimal ratios for composite amendments can significantly enhance the efficiency of remediating heavy metal-contaminated soil.

#### 3.1.2. Inorganic Solid Waste 

Inorganic solid waste can be categorized into six main groups based on their origin, such as the electric power industry, non-ferrous metal mining industry, thermal industry, metal processing and smelting industry, paper printing industry, and other industries. Table 7 shows the classification of inorganic industrial solid waste.

Inorganic solid waste is primarily used in soil as an amendment or stabilizer, offering solidification and stabilization effects. These wastes include stabilization and solidification agents. Stabilization agents reduce heavy metal mobility and solubility in contaminated soil without altering soil particle strength or permeability significantly. Solidification agents bind contaminated soil particles, forming a strong, low-permeability solid mass, effectively fixing heavy metals [64].

**Figure 8:**
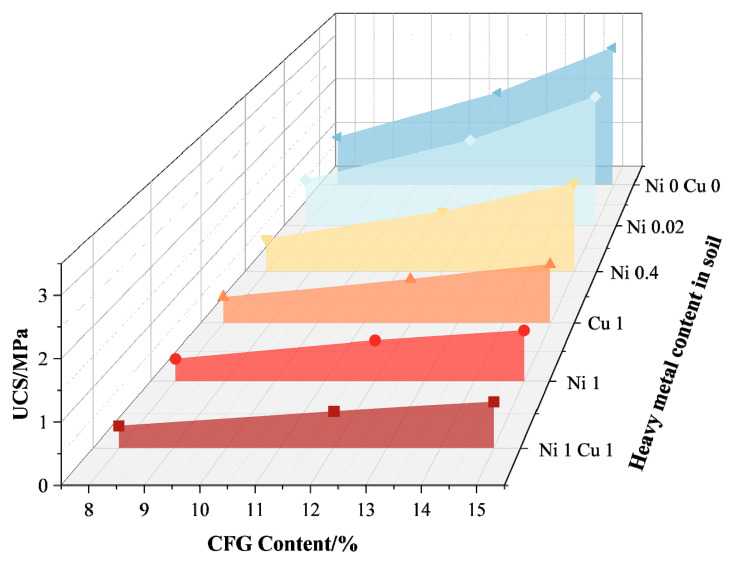
The relationship between curing agent content and UCS [26].

**Figure 9:**
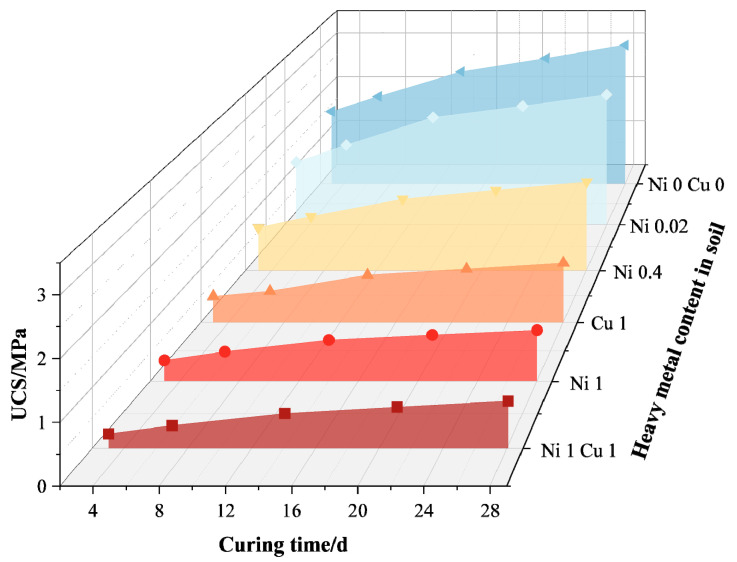
The relationship between curing time and UCS [26].

Inorganic solid waste amendments commonly used for soil improvement include carbide slag, fly ash, and cement. The remedial effects of inorganic solid waste amendments on heavy metal-contaminated soil are primarily evident in the following three aspects: (1) Improving the physical and chemical properties of the soil. Sun et al. [41] found that incorporating carbide slag into copper (Cu)-contaminated soil can increase the unconfined compressive strength (UCS) and pH value, which helps alleviate soil acidification. Wang et al. [26] investigated the effect of a new composite cementitious material consisting of cement, fly ash, and desulfurization gypsum (CFG) on the compressive strength and impermeability of contaminated soil containing different concentrations of nickel (Ni) and Cu. The ratios of total Ni content to dry soil mass used in this study were 0%, 0.02%, 0.4%, and 1%, while the ratios of total Cu content to dry soil mass were 0% and 1%, resulting in six heavy metal levels: Ni 0 Cu 0, Ni 0.02, Ni 0.4, Ni 1, Cu 1, and Ni 1 Cu 1. Figure 8 and Figure 9 show that unconfined compressive strength (UCS) decreases with increasing heavy metal concentration. Figure 8 indicates that CFG content between 8% and 15% enhances soil strength by forming gel-like hydrates that fill soil pores. Figure 9 illustrates the long-term benefits of CFG for the solidification of Ni-Cu-contaminated soils. The study conducted by Chang et al. [64] utilized three different leaching tests to determine the concentration of Cr in the leachate of cement powder and cement mortar at various substitution levels. The results of the experiment showed that cement mortar can reduce the leaching of Cr through the hydration process and enhance its encapsulation. Co-processing in cement kilns is an effective alternative method for the remediation of heavy metal-contaminated soil. 

(2) Increasing the number of soil microorganisms and enhancing enzyme activity. Fly ash, a byproduct of coal-fired power plants, has traditionally been considered a problematic solid waste. However, some studies have suggested that fly ash can be used as a soil amendment to improve the physical, chemical, and biological properties of degraded soil and serve as a source of both micro and macro-nutrients for plants [65]. Fly ash contains trace amounts of toxic elements and heavy metals, with aluminum (Al) mostly bound in insoluble aluminosilicate structures, which limits its biological toxicity. Additionally, fly ash contains macro and micronutrients, making it an effective soil amendment for improving soil health and crop yield [55]. For instance, Nayak et al. [55] conducted a pot experiment to investigate the effects of fly ash application on microbial reactions, soil enzyme activity, and heavy metal accumulation in soil and rice. The experiment found varying microbial responses to fly ash application rates. Fungi and actinomycetes decreased with higher fly ash rates, while aerobic heterotrophic bacteria remained stable until a 40% fly ash application. Total microbial activity and denitrifies increased initially but plateaued after 40%. Alkaline and acid phosphatase activities decreased with fly ash. Applying 10–20% fly ash by soil volume can boost trace elements, microbial activity, and crop yield. However, high fly ash levels may induce oxidative stress in rice plants, mitigated by antioxidants and enzymes. Lower fly ash doses show promise in enhancing methane-producing bacteria and rice yields in nutrient-poor soils [66]. (3) The application of ash-fly ash mixture (AFAM) improves soil fertility by improving soil structure and increasing porosity and water retention. These mixtures also act as liming agents, neutralizing acidic soils and making essential nutrients more accessible. In addition, they provide plant-friendly micronutrients while immobilizing heavy metals, thereby reducing their bioavailability and potential toxicity in the soil [67].

However, there are limitations in the use of inorganic solid waste in the process of remediating heavy metal-contaminated soil. For example, fly ash has a strong adsorption and fixation effect on the added phosphorus (P), which leads to low effectiveness of P in fly ash [68]. Additionally, the high effectiveness of boron (B) in fly ash is detrimental to crop growth. Applying high doses of fly ash can worsen heavy metal pollution and hinder microbial activity. Therefore, adjusting the proportion of fly ash application can help avoid adverse effects on crops [65]. The proportion of inorganic solid waste application significantly affects the remediation effect. Applying high doses of fly ash can lead to the accumulation of toxic metals in the soil and inhibit the growth and activity of microorganisms [55].

### 3.2. Organic Soil Amendments

#### 3.2.1. Organic Solid Waste 

Organic solid waste can include sewage sludge, municipal solid waste, food waste, kitchen waste, garden waste, agricultural waste, and animal waste [69]. Organic solid waste consists of biodegradable organic matter with a water content below 85–90%. Waste like paper sludge, sewage sludge, urban solid waste, crop straw, green manure, and animal manure can serve as organic soil amendments, particularly useful in addressing heavy metal pollution. The role of organic solid waste in improving the remediation of heavy metal-contaminated soil can be observed in several aspects. (1) It improves the physical and chemical properties of the soil. Municipal solid waste compost and cow manure are commonly used as organic fertilizers in agricultural production and horticultural cultivation. Studies have shown that under greenhouse conditions, these two types of organic solid waste significantly decrease the soil pH and increase the electrical conductivity (EC), which depends on the raw materials and ion concentrations of the compost [70,71]. (2) It reduces the bioavailability of heavy metals in the soil. Research conducted by Alam et al. found that a mixture of vermicompost (VC), leaf compost (LC), and spent mushroom compost (SMC) in equal proportions can raise the pH of heavy metal-contaminated soil, thereby reducing the bioavailability and mobility of heavy metals in the soil [27,72]. This effectively decreases the absorption of Cd, Cr, Pb, and Mn by plants, promoting plant growth [27]. (3) It improves the biological characteristics of contaminated soil. Incorporating straw into the soil enhances its biological characteristics by altering the bacterial and fungal community structure and increasing the biomass of bacteria and fungi. This, in turn, accelerates straw decomposition [73]. Additionally, the activities of soil phosphatase, urease, and transformation enzymes increase with the application of straw incorporation [74]. Furthermore, the application of biogas residue and compost improves several soil microbial characteristics, such as substrate-induced respiration, potential ammonium oxidation, and nitrogen mineralization [75]. Research has also shown that sewage sludge can serve as a barrier for heavy metal pollutants in solid waste landfill sites, hindering the ion migration of heavy metal pollutants [58]. The relationship between ion strength and electrical conductivity has been summarized by Griffin and Jurinak [76] with a correlation coefficient of 0.996.

(4) I=0.013EC

In the equation, I (mol/L) represents ionic strength, and EC (mmhos/cm) represents conductivity.

The compacted sewage sludge hinders the migration of heavy metal ions due to the anaerobic neutral to weakly alkaline conditions caused by microbial respiration [58]. (4) Furthermore, it enhances soil fertility and promotes plant growth. Green manure, as a main organic fertilizer, has been widely proposed as a sustainable method for ecological restoration and improving soil fertility in degraded environments [77]. Leguminous green manure is an important source of carbon (C) and nitrogen (N) in the cropping system, and the biochar derived from leguminous green manure can be used as a soil amendment to enhance soil fertility [23]. In addition, the organic acids released during straw decomposition can activate inorganic phosphorus, making it a new source of nutrients [78].

Although organic solid waste can improve heavy metal-contaminated soil, it also has some negative impacts. Chen et al. [23] found that leguminous green manure can quickly release N through rapid turnover, but it limits the effectiveness of maintaining organic carbon in the soil. In addition, the high carbon-to-nitrogen ratio of sewage sludge can increase the extractable content of Zn, Cu, Cr, and Cu in the soil. Moreover, untreated sludge contains numerous pathogens, parasites, and toxic substances, which can have varying degrees of toxic effects on plants and organisms in the soil. When applying straw, attention should be paid to the dosage, as excessive straw can lead to nitrogen deficiency in crops; hence, it is best to use it in combination with nitrogen fertilizer.

#### 3.2.2. Naturally Extracted Polymer Compounds

Naturally extracted polymer compounds (NEPCs) are high molecular weight substances obtained from natural sources or minerals by chemical processes involving biochemical or photosynthetic mechanisms. Common NEPCs used to amend heavy metal-contaminated soils include lignin, cellulose, and chitin. 

Derived from plant biomass, particularly fibers and wood pulp, lignin is a byproduct of industries such as paper and wood. It shows significant potential in improving heavy clay and sandy soils. Lignin, a major component alongside cellulose in plant tissues, consists of hydrophilic and hydrophobic groups, contributing to its high organic molecular weight. Studies by Cai et al. [79] have investigated lignin-treated sludge, evaluating parameters such as particle size distribution, Atterberg limits, compaction characteristics, unconfined compressive strength, pH levels, and resistivity. Enhanced soil performance attributed to lignin treatment is linked to the formation of a more stable soil structure through cation exchange and lignin bonding. Lignin’s ability to fill soil pores and improve strength, permeability, and durability is facilitated by hydrolysis reactions, ion exchange mechanisms, and electrostatic forces [79,80,81,82].

Similarly, cellulose, a polysaccharide found in plant cell walls, enhances soil structure and moisture retention, benefiting plant growth [83,84,85,86]. Chitin, a polymer found in fungal cell walls and exoskeletons of insects and crustaceans, contributes to soil fertility and biological activity, offering potential in sustainable agriculture practices [28,87,88,89].

These NEPCs collectively contribute to (1) improvement in soil properties—enhancing physical, mechanical, and resistivity characteristics; (2) reduction in heavy metal ion concentrations—immobilizing heavy metal ions in the soil, thereby reducing their availability and toxicity; (3) additionally, it can change the soil’s microbial communities, enhancing their ability to suppress harmful microorganisms; (4) improving the carbon-to-nitrogen ratio in the soil enhances plant growth and increases crop yield. However, lignin, cellulose, and chitin can also have different negative effects on contaminated soils. For example, lignin and cellulose have complex structures and are more difficult for microorganisms to degrade, leading to their persistence in the soil [81,82,85,86]. Decomposition of chitin may increase soil acidity, interfering with plant growth and soil microbial activity [28,88]. Therefore, further research and trials are needed on the dosage and ratios of amendments to be used.

#### 3.2.3. Organic Material

Biochar is a material rich in organic carbon that is produced by the pyrolysis of agricultural biomass waste, such as wood chips or crop straw, in a limited oxygen environment [29]. Numerous studies have confirmed that biochar can immobilize heavy metals in the soil and reduce their accumulation in plants. This is mainly attributed to the high porosity, active functional groups, high pH value, and cation exchange capacity (CEC) of biochar [29,30,90,91]. Additionally, the application of biochar and modified biochar in the remediation of heavy metal-contaminated soil has shown good economic benefits [92,93]. For example, biochar made from sludge can not only reduce the huge treatment costs of urban sludge but also recover energy through further processing (such as sludge pyrolysis), promoting sustainable development [94]. Biochar can serve as an organic amendment for heavy metal-contaminated soil, with the following specific functions: (1) improving the physical and chemical properties of the soil. Biochar application can positively influence soil thermal properties by enhancing soil water retention and increasing soil moisture content [95]. Furthermore, biochar is an alkaline material that can increase the pH value of acid-contaminated soil [96]. Wang et al. [97] conducted pot experiments and found that wood-based biochar, bamboo-based biochar, straw-based biochar, and walnut shell-based biochar all significantly improved soil electrical conductivity. The pH and cation exchange capacity (CEC) of soil are interrelated. Biochar composites raise the soil pH, increasing CEC and improving binding with the negatively charged functional parts of organic matter [94]. Githinji and Berihun [98,99] found that the application of biochar to the soil significantly reduced soil bulk density and increased total porosity, soil pH value, total nitrogen, soil organic carbon, and available phosphorus and potassium (K) through experiments. (2) Adsorbing heavy metals in the soil and reducing their accumulation in plants. Biochar’s complex humic substances and mineral oxides co-precipitate, creating inner-sphere complexes that exchange heavy metals with cations like Ca^2+^ and Mg^2+^. Biochar’s adsorption on heavy metal-contaminated soil may vary depending on soil type and cation presence [91]. Bian et al. [29,30] conducted a study on rice fields contaminated with heavy metals, where they applied a soil amendment called biochar. After using biochar, the soil’s pH and total organic carbon content increased significantly and stayed higher. This helped immobilize Cd and Pb in the soil, as Al, iron (Fe), and phosphorus (P) minerals combined with the contaminated biochar particles. Kim et al. [100] investigated the effect of biochar on heavy metal content in paddy soil and the accumulation of heavy metals in rice through a pot experiment. Their research demonstrated that mineral components such as phosphate and carbonate in biochar can precipitate together with heavy metals. Park et al. [101,102] found that peat moss biochar reduced the mobility and bioavailability of Cu, Cd, and Pb through coordination between metal electrons and chemical bonds. The presence of functional groups in the biochar and their affinity to bind with heavy metal ions lowered their bioavailability. (3) Biochar has an impact on soil microbial populations and activity. Some studies have shown that the dosage of biochar affects the abundance and activity of soil microbial species. At a low concentration of 1% biochar, the relative abundance of bacteria and fungi species increases, while at a higher application rate of 5%, their abundance decreases [103]. The application of biochar to soil can further alter the composition of microbial communities and increase diversity, thereby stimulating specific microbial processes [104]. This enhances soil biochemical cycling through the interaction between rhizobia and bacteria, promoting nutrient absorption and crop yield [93]. (4) Moreover, biochar has been found to promote plant growth and increase crop yield. Wang et al. [97] conducted a pot experiment and found that, apart from bamboo biochar, all other types of biochar increased the dry weight of bamboo compared to the control group. Abulaiti et al. [105] applied biochar to rice fields and observed a significant increase in the net photosynthesis rate, transpiration rate, and grain yield of rice.

Biological charcoal makes significant contributions to improving heavy metal-contaminated soil. However, it is important to consider the application dosage. Research shows that as the dosage of biological charcoal increases, the adsorption of nitrogen by biological charcoal can lead to a decrease in available nitrogen content in the soil, affecting the soil’s nutritional status and thus reducing plant growth rate [90].

## 4. Synthetic Soil Amendments

Synthetic soil amendments are large organic polymers designed to mimic natural amendments. Research on these synthetics, like polyacrylamide and polyvinyl alcohol resin, is a focal point in both domestic and international studies [10]. Among them, polyacrylamide (referred to as PAM) is a widely used artificial synthetic amendment in recent years. Its main mechanisms for soil improvement are as follows: (1) improving the physical properties of the soil. PAM is a water-soluble synthetic polymer composed of repeat units with amide functional groups. The amide groups are non-ionizable, but by introducing anionic or cationic functional groups, the PAM macromolecules can carry charges and form the ionic form of the polymer [106]. Rabiee et al. [107] used free radical polymerization to produce anionic PAM with metal cations and found that anionic PAM can form a composite material with soil structure, enhancing the adhesive force between soil and polymer functional groups, agglomerating soil components, and increasing soil strength. Sandy soils contaminated with heavy metals have poor water-holding capacity, and water and nutrients are easily lost [108]. PAM notably boosted the slope of the soil moisture curve’s inflection point, enhancing water retention and decreasing hydraulic conductivity in sandy soil, thus improving its physical properties. Additionally, PAM reduces soil salinity and bulk density while increasing organic matter, water-stable aggregates, and macroaggregate content [105]. (2) Adsorb heavy metals and reduce the bioavailability of heavy metals to organisms. Wiśniewska and Fijałkowska et al. [106] demonstrated through electrokinetic and potentiometric titration measurements by analyzing the solution pH, mineral type, different ionic types of PAM, properties of heavy metal ions, and the order of adsorbent addition, that anionic PAM and cationic PAM have different adsorption capacities at different pH values. Both anionic PAM and cationic PAM can immobilize Pb and Cr on the surface of clay minerals [31]. Superabsorbent polymers are loosely cross-linked polymer chain networks with high hydrophilicity, which can absorb and retain water or aqueous solutions up to several hundred times their weight [109]. Applying PAM to soil can reduce the uptake of Cr, Cu, Pb, and Zn by potato (*Solanum tuberosum* L.) plants irrigated with wastewater [109,110]. (3) Influence the activity of microorganisms and enzymes in the soil. PAM can affect the growth and respiration of microorganisms by lowering the soil pH and altering the chemical properties of the soil. PAM can be decomposed by some microorganisms in the soil, such as Bacillus subtilis, as a source of carbon and nitrogen, promoting microbial reproduction and increasing their population [111]. (4) Reduce the loss of nitrogen in the soil and increase crop yield. PAM can also increase the net photosynthetic rate, transpiration rate, and stomatal conductance of rice, positively impacting rice yield [105].

However, PAM has limitations as a soil amendment for heavy metal pollution. Its intermediate product, acrylamide, is toxic, and excessive use may lead to secondary pollution in heavy metal-contaminated soil. Further research is needed to determine the appropriate application rate of PAM.

## 5. Natural-Synthetic Copolymer Soil Amendments

Natural-synthetic copolymers like humic acid-polyacrylic acid, cellulose-polyacrylamide, and others can be used to remediate heavy metal-contaminated soil [32,85,112,113,114,115]. Due to their high molecular weight and numerous functional groups, natural-synthetic copolymer amendments play an important role in absorbing heavy metal elements. Thus, polymer-based nanocomposites can be used to immobilize metal ions in contaminated soil [32,85,112,113,114,115].

**Figure 10:**
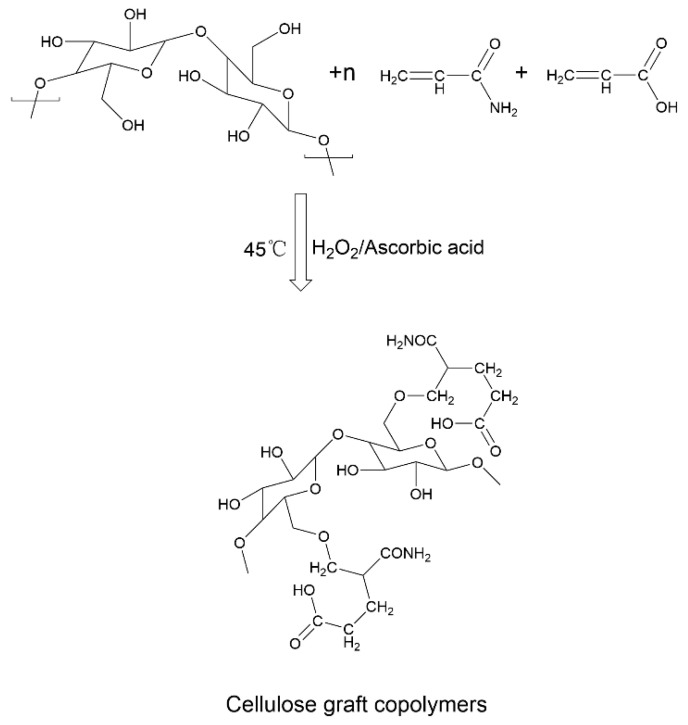
Reaction steps for the synthesis of cellulose graft copolymers [85].

The main improvements of natural-synthetic copolymer soil amendments on heavy metal-polluted soil can be summarized as follows: (1) adsorption and fixation of heavy metals in the soil, reducing the effective accumulation of heavy metals in the soil and plants. Mahdavi et al. [116] developed a novel and inexpensive polymer nanocomposite material called polyacrylamide-polyvinyl styrene/montmorillonite. This modifier is prepared by in situ polymerization of acrylamide and styrene between the interlayers of montmorillonite through free radical polymerization [116]. Guleria et al. [85] developed a cellulose-g-poly(acrylamide-co-acrylic acid) copolymer and tested its strong adsorption capacity for Cd, Cu, Pb, and Zn by varying pH, contact time, temperature, and metal ion concentration. The reaction steps for synthesizing cellulose-grafted copolymers are shown in Figure 10.

Zhou et al. [113] prepared polyacrylamide/montmorillonite by solution polymerization of acrylamide on γ-methacryloxypropyltrimethoxysilane-modified montmorillonite. Chemical leaching is a remediation technique for heavy metal-polluted soil using chelating agents to leach the contaminated soil [1]. However, chelating agents used in chemical leaching may cause secondary pollution to the soil. Therefore, Zhao et al. [112] studied a biodegradable lignin-based poly(acrylic acid) composite material (LBPAA). It can transfer Cd^2+^ and Pb^2+^ ions from the soil to the eluent, making it a new environmentally friendly composite material. (2) Improve the physical properties of soil to enhance its water retention and soil conservation ability. Li et al. [32] developed a multifunctional microsphere heavy metal-contaminated soil amendment called chitosan-grafted poly(acrylamide-co-acrylic acid)/biochar (CPB). CPB greatly enhances the soil’s ability to adsorb heavy metals. Its dense porous structure, which includes N-methylene bisacrylamide and hydrophilic biochar, reduces water loss, improves water retention, and enhances soil’s physical properties. Basuki et al. [114] used gamma rays to synthesize soil amendments by grafting chitosan-acrylamide copolymer, and the research showed that this amendment reduced the bulk density of soil, enhanced its water retention capacity, and increased porosity. (3) Control and release nitrogen sources in soil. Niu and Li [115] prepared starch-g-poly(vinyl acetate) (St-g-PVAc), which is a biodegradable carrier material through graft copolymerization of starch and vinyl acetate. It exhibits relatively low swelling, high encapsulation capacity, and a slow release rate.

## 6. Biological Soil Amendments

Bioremediation is a great option for fixing heavy metal-contaminated soil. It uses microbial amendments to remove heavy metals, offering economic and ecological benefits with minimal negative impacts [1,117]. Microbial remediation of heavy metal-contaminated soil occurs through the biological activity of microorganisms, which adsorb or transform heavy metals into less toxic products [117]. This process primarily involves mechanisms such as biosorption, biovolatilization, bioleaching, bioaccumulation, and biomineralization [118]. 

Bioremediation agents are suitable for repairing large areas with relatively low pollutant concentrations in shallow sites. The main effects of bioremediation are: (1) improving the physical and chemical properties of soil. Liu et al. [119] added feces to oil-contaminated soil to biostimulate the local microorganisms for bioremediation. The use of the *Pseudomonas chenduensis* strain MBR for microbial remediation of heavy metal-contaminated soil demonstrated that adding MBR can improve the total phosphorus, total carbon, total nitrogen, and pH value of the soil while also increasing the dry weight of grains [33]. (2) To reduce the overall bioavailable fraction of heavy metals in contaminated soil and decrease their toxicity, microorganisms can transform heavy metals from one oxidation state or organic compound to another [12]. *Aspergillus niger* produces metal-solubilizing organic acids, which can leach heavy metals like Cu, Cd, Pb, and Zn from the soil, removing their exchangeable forms and other compounds. Microbial leaching for bioremediation is more effective and cost-efficient than chemical leaching [120]. Peng et al. [13] used *Rhodobacter* bacteria to decrease the bioavailable fractions of Cd and Zn in their exchangeable and carbonate-bound phases while increasing the more stable forms, such as iron/manganese oxides and organic complexes. After wheat seedling experiments with *Fusarium solani*, Cd uptake by plants decreased, and exchangeable Cd and Zn in contaminated soil dropped by 30.7% and 100.0%, respectively. Li et al. [33] used *Pseudomonas chenduensis* strain MBR (referred to as strain MBR) to bioremediate Cd-contaminated paddy soil, and the results showed that the addition of strain MBR enhanced the role of the microbial community in transforming Cd components, significantly reducing Cd accumulation in rice grains and roots. (3) Increasing the quantity of microorganisms in the soil promotes the diversification of soil microbial communities. Liu et al. [119] conducted experiments to demonstrate that soil treated with microbial remediation had significantly increased numbers of heterotrophic bacteria, polycyclic aromatic hydrocarbon-degrading bacteria, and total petroleum hydrocarbon-degrading bacteria. The microbial activity, species, and diversity were all enhanced. (4) Enhancing plant growth and making plants more robust can increase their resistance to pathogenic bacteria. Basyal and Emery [121] conducted in-depth research on the plant-arbuscular mycorrhizal fungi (AMF) symbiotic system and found that AMF can increase leaf number, root biomass, cellulose, and hemicellulose content in plants. This promotes plant growth and improves key indicators related to plant growth and cell wall chemistry, with the most significant effects observed under low soil moisture conditions.

Biological agents have limits in remediation. Microbes can change heavy metals into less toxic forms but cannot remove them from the soil, requiring additional extraction methods [12]. Additionally, microbial remediation is influenced by environmental conditions, requires extended remediation periods, and is not suitable for soils with high contamination levels or low permeability [117]. 

## 7. Conclusions and Recommendations

This review synthesizes recent research on soil amendments for mitigating heavy metal contamination, emphasizing their impacts on soil properties, heavy metal concentrations, microbial communities, and crop yields. The selection criteria for amendments are based on their efficacy, application timing, cost-effectiveness, and ecological advantages. Soil amendments for heavy metal-contaminated soils exhibit several key effects: (1) They enhance soil structural integrity, improve water retention and nutrient holding capacity, modify pH levels, and ameliorate other physicochemical properties. (2) They diminish the availability and mobility of heavy metals such as Cu, Ni, Cd, Cr, Pb, Zn, and Co by converting them into less toxic forms, thereby reducing their uptake by plants. (3) They stimulate soil microbial populations, modify microbial community composition and structure, increase community diversity, and boost enzyme activity. (4) They enhance soil fertility, adjust carbon-to-nitrogen ratios, promote plant growth, and subsequently increase crop yields.

There is a notable deficiency of multifunctional, cost-effective, and environmentally benign soil amendments for heavy metal-contaminated soils. The limitations of current approaches are as follows: (1) Natural mineral amendments are constrained by their limited effectiveness and availability, resulting in suboptimal efficiency. (2) Amendments derived from inorganic and organic solid wastes may introduce secondary pollution and inhibit microbial and enzymatic activities due to challenges in dosage control. (3) Artificially synthesized amendments are expensive to produce, and some intermediate products present potential pollution risks. (4) Biological amendments are unable to completely eliminate heavy metals and exhibit delayed effectiveness over extended testing periods. (5) While natural-synthetic copolymer amendments demonstrate high efficiency, further research is required to elucidate their synthesis, underlying mechanisms, and economic feasibility.

Based on the applicability and limitations of the aforementioned soil amendments, we propose the following recommendations: (1) Investigate the mechanisms and impacts of various soil amendments on heavy metal-contaminated soils, with consideration given to pollution control requirements and cost factors associated with industrial production, transportation, and agricultural application. Emphasize the development of cost-effective and environmentally friendly amendments while prioritizing both cost reduction and environmental protection. (2) Relying on a single amendment may yield limited effectiveness. It is advisable to explore combinations of amendments, such as mixing inorganic-organic solid wastes or integrating biological amendments with agricultural and industrial byproducts. Combining different types of amendments can address the limitations of individual treatments. Employing orthogonal experimental designs can assist in identifying optimal combinations and proportions to enhance effectiveness and functionality. (3) While current research predominantly focuses on soil physicochemical properties, heavy metal removal, biological characteristics, and crop yield improvements, further investigation is warranted into the changes in soil properties and biological dynamics before and after remediation, as well as the mechanisms of soil–plant interactions. (4) The effectiveness of soil amendments for remediating heavy metal-contaminated soils may vary across different climates and terrains. Most studies are conducted in laboratory settings, small plots, or greenhouses, which may not accurately predict performance in larger outdoor environments. It is crucial to undertake more large-scale field experiments to better understand the real-world effects of these amendments. (5) Ensure the maintenance of remediated soils, establish comprehensive soil assessment systems, and preserve ecosystem functions to promote sustainable ecological, economic, and social benefits.

## Figures and Tables

**Table 1:** Soil remedies selected most frequently in recent decision documents (FY 2018–2020) [19].

Selected Remedy	Number	Percent
Treatment	48	37%
In Situ Treatment	37	28%
Thermal Treatment	14	11%
Soil Vapor Extraction	13	10%
Solidification/Stabilization	8	6%
Chemical Treatment	5	4%
Bioremediation	3	2%
Flushing	2	2%
Multi-phase Extraction	2	2%
Soil Amendments	2	2%
Ex Situ Treatment	17	13%
Solidification/Stabilization	7	5%
Physical Separation	6	5%
Thermal Treatment	2	2%
Containment/Disposal	90	69%
Monitored Natural Attenuation	1	1%
Institutional Controls	98	75%
Other	21	16%

**Table 2:** Some typical soil amendment samples are used for the remediation of heavy metal-contaminated soils.

Soil Amendments	Classification	Experiment Types	Heavy Metals	Remediation Effectiveness	Reference
Vermiculite	Natural mineral	Pot experiments	Cu, Cr, Ni	Significantly reduce the absorption of metal pollutants by mustard and spinach plants.	[25]
Cement, Fly ash, Desulfurization gypsum	Inorganic solid waste	Solidification/ Stabilization	Cu, Ni	Significantly increase the compressive strength and permeability of contaminated soils.	[26]
Vermicompost, Leaf compost, Spent mushroom compost	Organic solid waste	Greenhouse experiments	Cd, Cr, Pb, Mn	Decrease the absorption of Cd, Cr, Pb, and Mn by plants, promoting plant growth.	[27]
Lignin, Chitin	Naturally extracted polymer compounds	Kinetics experiments, Adsorption experiments	Cu, Fe	Show high adsorption capacity for metal ions, especially at low concentrations.	[28]
Biochar	Organic material	Field experiments	Cd, Pb	Significantly increase the pH value and total organic carbon content of the soil and effectively immobilize Cd and Pb in the soil.	[29,30]
Polyacrylamide	Synthetic	Adsorption experiments	Pb, Cr	Immobilize Pb and Cr on the surface of clay minerals.	[31]
Chitosan-grafted poly(acrylamide-co-acrylic acid)/biochar	Natural-synthetic copolymer	Kinetics experiments, Adsorption experiments	Cu	Effectively increase the adsorption capacity of soils for heavy metals and improve the water retention of soils.	[32]
*Pseudomonas chenduensis*	Biological	Pot experiments	Cu, Cd, Pb, Zn	Enhance the role of the microbial community in transforming Cd components and reduce Cd accumulation in rice grains and roots.	[33]

**Table 3:** The top 10 countries in terms of publication volume.

Ranking	Count	Centrality	Countries
1	155	0.57	People’s Republic of China
2	35	0.64	Spain
3	25	0.27	USA
4	21	0.17	France
5	20	0.25	Australia
6	18	0.1	Pakistan
7	16	0.06	South Korea
8	15	0.01	Italy
9	11	0.21	Czech Republic
10	11	0.01	Poland

**Table 4:** The top 10 most occurring keywords.

Ranking	Count	Centrality	Keywords
1	191	0.02	Heavy metal
2	105	0.07	Stabilization
3	79	0.04	Fly ash
4	78	0.11	Immobilization
5	59	0.07	MSWI fly ash
6	57	0.13	Stabilization/solidification
7	53	0.15	Solidification/stabilization
8	49	0.11	Cement
9	44	0.15	Portland cement
10	40	0.09	Behavior

**Table 5:** The top 12 keywords with the strongest citation bursts.

Keywords	Year	Strength	Begin	End	2013–2023
organic amendment	2013	4.34	2013	2018	▃▃▃▃▃▃ ▂▂▂▂▂
mine tailing	2013	3.03	2013	2017	▃▃▃▃▃ ▂▂▂▂▂▂
black carbon	2014	2.93	2014	2019	▂ ▃▃▃▃▃▃ ▂▂▂▂
pollution	2021	2.48	2021	2023	▂▂▂▂▂▂▂▂ ▃▃▃
health risk	2021	2.38	2021	2023	▂▂▂▂▂▂▂▂ ▃▃▃
trace element	2017	2.37	2017	2018	▂▂▂▂ ▃▃ ▂▂▂▂▂
impact	2019	2.22	2019	2020	▂▂▂▂▂▂ ▃▃ ▂▂▂
plant	2019	2.22	2019	2020	▂▂▂▂▂▂ ▃▃ ▂▂▂
risk assessment	2017	2.1	2017	2019	▂▂▂▂ ▃▃▃ ▂▂▂▂
fractionation	2014	2.05	2018	2019	▂ ▂▂▂▂ ▃▃ ▂▂▂▂
paddy soil	2020	2.01	2020	2023	▂▂▂▂▂▂▂ ▃▃▃▃
copper	2014	2	2014	2017	▂ ▃▃▃▃ ▂▂▂▂▂▂

**Table 6:** Keyword clusters analysis and feature information.

Cluster-ID	Count	Silhouette	Year	Top Terms (LLR)
0	47	0.685	2018	lead-zinc smelting slag; heavy metal; solid waste (SW); material characteristics; cementitious property
1	45	0.635	2017	electrolytic manganese residue; calorimetry; building materials; fly ash; APC residues
2	40	0.685	2020	red mud; sewage sludge; cotreatment NBSP; arsenic-laden spent media; environmental risk assessment
3	34	0.866	2014	calcining pretreatment; sulfates; pickling liquor; hazard-free treatment; Cu/Zn
4	34	0.714	2018	potentially toxic elements; cement; leaching pattern; hazardous waste management; immobilization mechanisms
5	31	0.783	2018	municipal solid waste; fly ash; MSWI fly ash; hazardous waste; arsenic contaminated soil
6	30	0.885	2015	tobermorite; air pollution control residues; heavy metal speciation; reconstructed slag; vitrification
7	24	0.639	2020	pre-treatment; cementitious materials; solidification and stabilization; cement kiln co-processing; XPS
8	23	0.782	2016	MSWI fly ash; compressive strength; stabilization; hydration products; uncertainty
9	18	0.793	2016	heavy metal immobilization; alkali-activated technology; nano-alumina; synergistic effect; gelation

**Table 7:** The classification of inorganic industrial solid waste.

Industry Source	Name of Solid Waste	References
Electric power industry	Slag, desulfurization gypsum, fly ash, etc.	[15,26,48,49]
Non-ferrous metal mining industry	Tailings, coal gangue, limestone, gypsum, etc.	[50,51,52,53]
Thermal industry	Fly ash, slag, dust, etc.	[15,48,52,54,55]
Metal processing and smelting industry	Blast furnace slag, steel slag, dust, sludge, etc.	[56,57,58,59]
Paper printing industry	Deinking residue, plastic debris, tailings, etc.	[50,60,61]
Other industries	Waste clay, nuclear waste residue, etc.	[62,63]

## Data Availability

The authors confirm that the data supporting the study is available within the article.
